# Microbiological profile of patients treated for postoperative peritonitis: temporal trends 1999–2019

**DOI:** 10.1186/s13017-023-00528-1

**Published:** 2023-12-19

**Authors:** Philippe Montravers, Nathalie Grall, Elie Kantor, Pascal Augustin, Kevin Boussion, Nathalie Zappella

**Affiliations:** 1 Department of Anaesthesiology and Surgical Intensive Care, DMU PARABOL, APHP, Hôpital Bichat, 75018 Paris, France; 2 UFR Paris Nord, Université Paris Cité, 75006 Paris, France; 3 INSERM UMR 1152 PHERE, Université Paris Cité, 75018 Paris, France; 4 INSERM UMR 1137 IAME, Université Paris Cité, 75018 Paris, France; 5 Department of Bacteriology, AP-HP, Hôpital Bichat, 75018 Paris, France

**Keywords:** Postoperative peritonitis, Multidrug-resistant bacteria, Empirical antibiotic therapy, Adequate empirical therapy, Optimal empirical therapy

## Abstract

**Background:**

Temporal changes in the microbiological resistance profile have been reported in several life-threatening infections. However, no data have ever assessed this issue in postoperative peritonitis (POP). Our purpose was to assess the rate of multidrug-resistant organisms (MDROs) in POP over a two-decade period and to analyse their influence on the adequacy of empirical antibiotic therapy (EAT).

**Methods:**

This retrospective monocentric analysis (1999–2019) addressed the changes over time in microbiologic data, including the emergence of MDROs and the adequacy of EAT for all intensive care unit adult patients treated for POP. The in vitro activities of 10 antibiotics were assessed to determine the most adequate EAT in the largest number of cases among 17 antibiotic regimens in patients with/without MDRO isolates. Our primary endpoint was to determine the frequency of MDRO and their temporal changes. Our second endpoint assessed the impact of MDROs on the adequacy of EAT per patient and their temporal changes based on susceptibility testing. In this analysis, the subgroup of patients with MDRO was compared with the subgroup of patients free of MDRO.

**Results:**

A total of 1,318 microorganisms were cultured from 422 patients, including 188 (45%) patients harbouring MDROs. The growing proportions of MDR *Enterobacterales* were observed over time (*p *= 0.016), including ESBL-producing strains (*p *= 0.0013), mainly related to *Klebsiella* spp (*p *< 0.001). Adequacy of EAT was achieved in 305 (73%) patients. Decreased adequacy rates were observed when MDROs were cultured [*p *= 0.0001 vs. MDRO-free patients]. Over the study period, decreased adequacy rates were reported for patients receiving piperacillin/tazobactam in monotherapy or combined with vancomycin and imipenem/cilastatin combined with vancomycin (*p *< 0.01 in the three cases). In patients with MDROs, the combination of imipenem/cilastatin + vancomycin + amikacin or ciprofloxacin reached the highest adequacy rates (95% and 91%, respectively) and remained unchanged over time.

**Conclusions:**

We observed high proportions of MDRO in patients treated for POP associated with increasing proportions of MDR *Enterobacterales* over time. High adequacy rates were only achieved in antibiotic combinations involving carbapenems and vancomycin, while piperacillin/tazobactam is no longer a drug of choice for EAT in POP in infections involving MDRO.

**Graphical Abstract:**

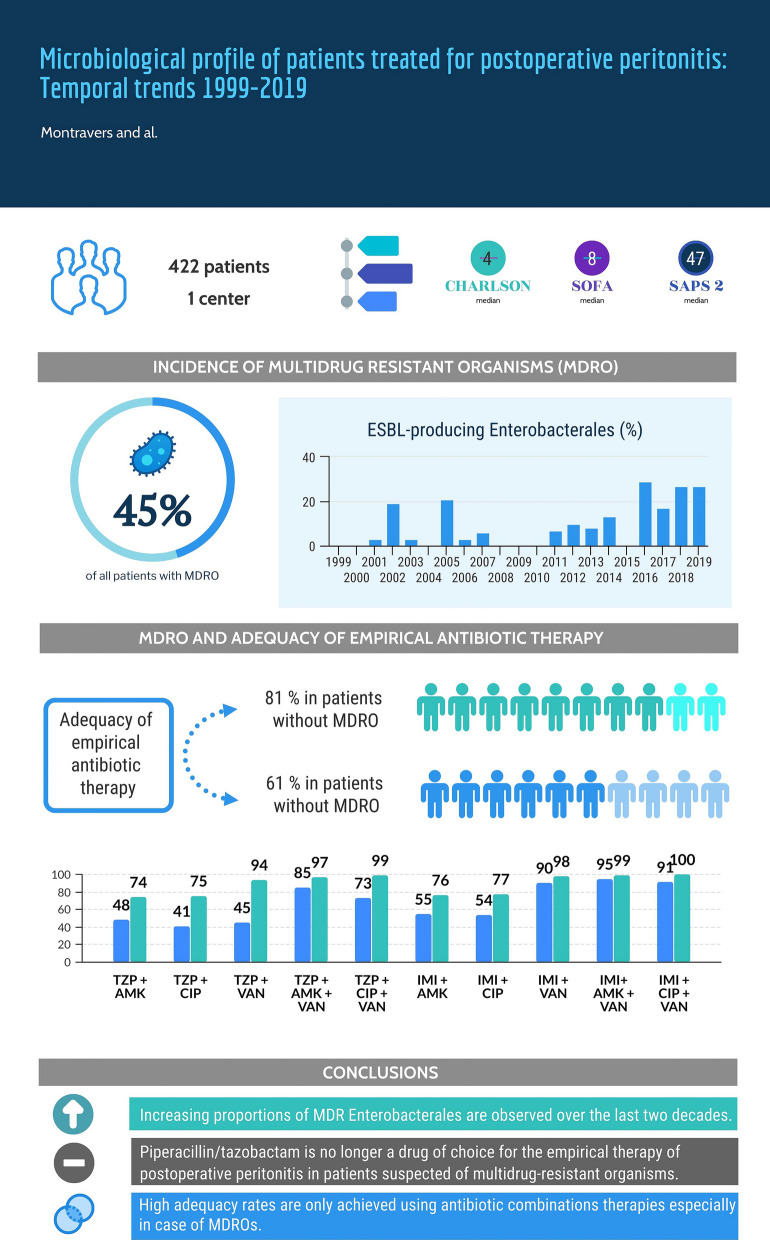

**Supplementary Information:**

The online version contains supplementary material available at 10.1186/s13017-023-00528-1.

## Introduction

Timely and adequate anti-infective therapy and source control are as key elements for improving the prognosis of life-threatening infections [1, 2]. Over the recent decades, increasing rates of multidrug-resistant organisms (MDROs) have been reported in healthcare-associated infections [3]. The rise of extended-spectrum beta-lactamase (ESBL) and the emergence of carbapenemase are of particular relevance among aerobic Gram-negative bacilli (GNB), but Gram-positive cocci (GPC) remain a source of concern [4].

The management of postoperative peritonitis (POP) is challenging due to its polymicrobial nature. The threat raised by MDROs in the selection of empirical anti-infective therapy (EAT) has been addressed in many recommendations. However, the last guidelines for intraabdominal infections (IAIs) were based on data published more than a decade ago [5–8]. While several reports have demonstrated temporal changes in the microbiological profile of patients treated for life-threatening infections, no data have ever addressed this issue in intensive care unit (ICU) patients treated for POP [9–11]. Recent papers have reported high proportions of MDRO, which could be associated with increased proportions of inadequate EAT [12, 13].

The aim of this pragmatic observational study was first to assess the frequency of MDROs in POP and its evolution over a two-decade period. Resistance features cannot be summarized as the emergence of MDROs, which led us to comprehensively address the temporal changes in the adequacy rate of EAT and to determine the best theoretical EAT over the study period.

## Materials and methods

### Study population

In this retrospective single-centre study (January 1999 to December 2019), all consecutive adult patients who underwent reoperation for a diagnosis of POP and needed ICU management were included. POP was defined as an intraperitoneal infection occurring after an initial abdominal surgery confirmed by macroscopic findings and positive peritoneal fluid culture yielding at least one microorganism (bacteria or yeast) at reoperation [14]. Only the first surgical reoperation was considered. Patients treated for POP without microbiological cultures, those with negative microbiological cultures, pure fungal infection, superinfection of acute pancreatitis, and mesenteric ischaemia without bowel perforation were excluded from the analysis.

The protocol was designed in accordance with national laws and approved by the institutional review boards, which waived the need for signed informed consent due to the observational and retrospective nature of the study (CEERB CHU Bichat, Paris-Diderot-University, Paris, France, agreement n°10–008). The collection of data was declared to the Commission Nationale de l’Informatique et des Libertés (CNIL, declaration number 1413211v1). The study was performed in accordance with the STROBE recommendations [15] (Additional file 1: Table S1).

### Collected data

Demographic data and comorbidities (according to the Charlson’s score [16]) were recorded. The characteristics of the initial surgery (anatomical site, emergency, wound classification), the administration of antimicrobial therapy, and the need for reoperation prior to the diagnosis of POP were collected.

At the time of reoperation for POP, the SAPS II score (Simplified Acute Physiologic Score) and SOFA score (Sequential Organ Failure Assessment) were calculated [17, 18]. Organ failure was defined as a SOFA organ score > 2 points. The source of contamination, its anatomical location, and the delay for reoperation since the initial surgery were assessed.

### Microbiological data

Peritoneal samples collected during reoperation were immediately sent to the microbiology laboratory. Samples were processed according to standard laboratory methods. After Gram-staining for direct examination, cultures were incubated for 48 h at 35 °C ± 2 under aerobic and anaerobic conditions for Gram-positive and negative aerobic bacteria, anaerobes, and fungi (Additional file 1: text). All morphologically distinct colonies were identified by standard bacteriologic techniques and tested for antibiotic susceptibility by the disc diffusion method according to the Antibiogram Committee of the French Society of Microbiology and the European Committee on Antimicrobial Susceptibility Testing (EUCAST) [19, 20]. The resistance mechanisms were detected according to the EUCAST recommendations. The detection of methicillin-resistant *Staphylococcus aureus* (MRSA) was performed by disc diffusion using cefoxitin. The presence of ESBL was detected using the double-disc synergy test. The presence of carbapenemase was suspected when organisms were not susceptible to ertapenem and/or imipenem and confirmed by PCR to have carbapenemase genes, including *bla*_VIM_, *bla*_KPC_, *bla*_OXA-48_, *bla*_NDM_, and *bla*_IMP_ (homemade PCR until 2014 and Xpert Carba-R kit (Cepheid, Sunnyvale, CA, USA) since 2014).

The susceptibility to ten antibiotics (amoxicillin/clavulanic acid; piperacillin/tazobactam; cefotaxime; ceftazidime; imipenem/cilastatin; ciprofloxacin; gentamicin; amikacin; vancomycin and metronidazole) was assessed and reported when available. The results were expressed as proportions of susceptible bacteria for each antibiotic.

MDR organisms were defined according to the criteria of the European Centre for Disease Prevention and Control (ECDC) as bacteria nonsusceptible to at least one agent in three or more antimicrobial categories [21]. The MDRO profile was assessed for *S. aureus* and *Enterococcus* spp. for MDR aerobic GPC and *Enterobacterales* (other than *Salmonella* and *Shigella*), *Pseudomonas aeruginosa* and *Acinetobacter* spp. for MDR aerobic GNB.

### Empirical antimicrobial therapy

EAT was started at the time of reoperation according to the French recommendations and our institutional protocol [7]. The selection of therapy was based on the severity of the case, previous antibiotic therapies, and local epidemiology. According to French guidelines, EAT is a combination of broad-spectrum beta-lactams, including piperacillin/tazobactam or imipenem/cilastatin (depending on initial severity), combined with aminoglycosides ± vancomycin when MRSA or amoxicillin-resistant enterococci are suspected [7]. EAT was deemed adequate when all the cultured bacteria were targeted by at least one administered antibiotic [14].

### Theoretical best empirical antibiotic regimen

The analysis of the regimens classified as monotherapy or combination therapy (two-, three-, and four-drug antibiotics) allowed the assessment of 17 theoretical regimens to achieve adequate EAT in the largest number of cases. These analyses were performed according to the presence or absence of MDRO. As the purpose of this study was to focus on antibiotic therapy, fungi were not included in the definition of adequacy. EAT was considered adequate if all the bacteria isolated from the surgical peritoneal samples were susceptible to at least one of the antibiotics used [14]. EAT was considered adequate or inadequate strictly on the basis of culture results.

### Statistical analysis

Our primary objective was to determine the frequency of MDRO and their temporal changes among patients who underwent reoperation for POP. Second, we assessed the impact of MDROs on the adequacy of EAT per patient and their temporal changes based on susceptibility testing. In this analysis, the subgroup of patients with MDRO was compared with the subgroup of patients free of MDRO.

The results were expressed per microorganism and per patient when clinical care was the key issue. Continuous variables were expressed as medians with interquartile ranges (IQRs) and were compared using Student’s t test or Wilcoxon’s rank sum test, as appropriate. Categorical variables were expressed as absolute numbers and proportions and were compared with Fisher’s exact test or the chi-square test, as appropriate. To assess the temporal trends of MDRO and EAT, the yearly changes were analysed using linear regression or the Cochran–Armitage test for trends in categorical variables.

Missing data were not replaced in the final dataset. As the study was strictly retrospective and observational, no power calculation was performed [22]. The statistical analyses were performed using JMP software (SAS Institute Inc., Cary, NC, USA). The significance threshold was a priori set at a significance of 0.05.

## Results

### Study population

Overall, 422 patients who underwent reoperation for POP were analysed (Additional file 1: Fig. S1). Their demographic characteristics, clinical data, and outcomes are presented in Table 1.

**Table 1:** Clinical characteristics and outcome of the study population

Variables	Overall *N *= 422 patients	Missing value	MDRO group *N *= 188 patients	MDRO-free group *N *= 234 patients	*p* value
*Demographic characteristics*					
Age—years, median [IQR]	61 [49–73]	0	64 [53–74]	59 [46–72]	0.0137
Patients aged ≥ 80 years—*n* (%)	45 (11)	0	24 (13)	21 (9)	0.208
Male sex—*n* (%)	221 (52)	0	115 (61)	106 (45)	0.0012
*Medical history and underlying diseases*					
No underlying disease—*n* (%)	9 (2)	0	4 (2)	5 (2)	1.000
Rapidly fatal underlying disease (survival < 1 year)—*n* (%)	111 (26)	0	58 (31)	53 (23)	0.057
Charlson score, median [IQR]	4 [1–6]	0	4 [2–6]	3 [1–5]	0.027
Diabetes mellitus*—*n* (%)	77 (18)	0	38 (20)	39 (17)	0.348
Malignancy*—*n* (%)	165 (39)	0	82 (44)	83 (35)	0.088
Chronic obstructive pulmonary disease*—*n* (%)	46 (11)	0	25 (13)	21 (9)	0.156
Moderate to severe chronic kidney disease*—*n* (%)	17 (4)	0	7 (4)	10 (4)	0.809
Immunosuppression—*n* (%)	75 (18)	0	40 (21)	35 (15)	0.091
*Initial surgery*					
Emergency surgery—*n* (%)	162 (38)	0	82 (44)	80 (34)	0.047
Contaminated or septic surgery—*n* (%)	159 (38)	0	79 (42)	80 (34)	0.098
Digestive surgery—*n* (%)	367 (87)	0	166 (88)	201 (86)	0.466
Bariatric surgery—*n* (%)	79 (19)	0	19 (24)	60 (76)	< 0.0001
Gynaecological surgery—*n* (%)	24 (6)	0	6 (3)	18 (8)	0.056
Curative antibiotic therapy—*n* (%)	116 (27)	0	55 (29)	61 (26)	0.054
Abdominal reoperation before diagnosis of POP—*n* (%)	114 (27)	0	53 (28)	61 (26)	0.625
Delay between initial surgery and reoperation for POP, days, median [IQR]	7 [4–12]	1	8 [4–13]	7 [4–11.5]	0.214
Antimicrobial therapy before reoperation for POP—*n* (%)	278 (66)	1	130 (69)	148 (63)	0.203
Ongoing antimicrobial therapy at the time of diagnosis of POP—*n* (%)	232 (56)	4	111 (60)	121 (52)	0.099
*Surgical management of POP*					
Colonic or rectal source of contamination—*n* (%)	119 (28)	0	71 (64)	48 (42)	0.0008
Small bowel source of contamination—*n* (%)	117 (28)	0	55 (56)	62 (53)	0.599
Gastroduodenal source of contamination—*n* (%)	99 (23)	0	28 (38)	71 (60)	0.004
Pancreas and biliary source of contamination—*n* (%)	23 (5)	0	13 (7)	10 (4)	0.281
Perforation—*n* (%)	124 (29)	0	57 (30)	67 (29)	0.705
Anastomosis dehiscence—*n* (%)	155 (37)	0	64 (34)	91 (39)	0.304
Abscess—*n* (%)	80 (19)	0	37 (20)	43 (18)	0.733
No cause—*n* (%)	67 (16)	0	26 (14)	41 (18)	0.302
Source control for POP					
Resection/excision—*n* (%)	292 (69)	4	128 (68)	164 (70)	0.717
Ostomy—*n* (%)	242 (57)	4	115 (61)	127 (54)	0.136
Anastomosis—*n* (%)	37 (9)	4	12 (4)	25 (11)	0.165
Patch/suture—*n* (%)	109 (26)	4	40 (21)	69 (29)	0.055
Intraoperative drainage—*n* (%)	351 (82)	4	156 (83)	195 (83)	1.000
*Severity criteria at the time of ICU admission for POP*					
SAPS II score, median [IQR]	47 [35–58]	1	50 [36–59]	45 [33–57.5]	0.059
SAPS II score ≥ 40—*n* (%)	281 (67)	1	110 (71)	171 (64)	0.160
SOFA score, median [IQR]	8 [5–10]	5	8 [5–10]	8 [4–10]	0.992
Respiratory failure—*n* (%)	184 (45)	16	79 (43)	105 (47)	0.485
Hemodynamic failure—*n* (%)	284 (70)	16	131 (72)	153 (68)	0.421
Renal failure—*n* (%)	109 (27)	17	42 (23)	67 (30)	0.115
*Outcome*					
Reoperation—*n* (%)	203 (48)	0	89 (47)	114 (49)	0.778
Duration of ICU stay of survivors—days; median [IQR]	14 [8–25]	0	16 [9–28]	12 [7–25]	0.080
Duration of mechanical ventilation—days; median [IQR]	7 [3–15]	0	7 [4–16]	6 [3–15]	0.214
Death in ICU—*n* (%)	134 (32)	0	64 (34)	70 (30)	0.365
Delay of death in the ICU—days; median [IQR]	14 [3–26]	0	12 [3–27]	15 [3–25]	0.675
Death in hospital—*n* (%)	148 (35)	0	76 (40)	72 (31)	0.038

*According to the Charlson’s comorbidity index

### Microbiological characteristics

A total of 1,318 microorganisms were isolated, including 88% polymicrobial cultures (Table 2 and Additional file 1: Table S2). The type and proportions of the cultured organisms remained unchanged over the study period (Additional file 1: Table S3). The susceptibility profiles of GPC, GNB, and anaerobic bacteria, including MDROs, are presented in Tables 3, 4, 5.

**Table 2:** Microbiological samples expressed as numbers of microorganisms and proportions in patients with and without MDRO

Microorganisms	Overall *N *= 1318 organisms	Missing value	MDRO group *N *= 632 organisms	MDRO-free group *N *= 686 organisms	*p* value
Total number of MDRO—*n* (%)	243 (18)	0	243 (38)	–	–
Gram-positive aerobic cocci—*n* (%)	503 (38)	0	214 (34)	289 (42)	0.002
MDR Gram-positive cocci—*n* (%)	42 (3)	0	42 (7)	–	–
*Streptococcus* spp.—*n* (%)	129 (10)	0	33 (5)	96 (14)	0.0001
*Enterococcus* spp.—*n* (%)	254 (19)	0	131 (21)	123 (18)	0.198
MDR Enterococci—*n* (%)	31 (2)	0	31 (5)	–	–
*Enterococcus faecalis*—*n* (%)	144 (11)	0	63 (10)	81 (12)	0.284
*Enterococcus faecium*—*n* (%)	70 (5)	0	48 (8)	22 (3)	0.0005
MDR *Enterococcus faecium*—*n* (%)	30 (2)	0	30 (5)	–	–
*Other enterococci*—*n* (%)	40 (3)	0	20 (3)	20 (3)	0.872
*Staphylococcus aureus*—*n* (%)	35 (3)	0	15 (2)	20 (3)	0.608
MDR *Staphylococcus aureus*—*n* (%)	11 (1)	0	11 (2)	–	–
Coagulase-negative staphylococci—*n* (%)	82 (6)	0	35 (6)	47 (7)	0.361
Miscellaneous Gram-positive organisms—*n* (%)	3 (0.2)	0	0	3 (0.4)	
Gram-negative aerobic bacilli—*n* (%)	505 (38)	0	287 (45)	218 (32)	0.0001
MDR Gram-negative aerobic bacilli—*n* (%)	201 (15)	0	201 (32)	–	–
*Enterobacterales*—*n* (%)	426 (32)	0	237 (38)	189 (28)	0.0001
MDR *Enterobacterales*—*n* (%)	172 (13)	0	172 (27)	–	–
ESBL-producing *Enterobacterales*—*n* (%)	34 (3)	0	34 (5)	–	–
*Escherichia coli*—*n* (%)	200 (15)	0	118 (19)	82 (12)	0.0007
MDR *Escherichia coli*—*n* (%)	96 (7)	0	96 (15)	–	–
*Klebsiella* spp.—*n* (%)	59 (4)	0	29 (5)	30 (4)	0.694
MDR *Klebsiella* spp.—*n* (%)	19 (1)	0	19 (3)	–	–
*Enterobacter* spp.—*n* (%)	78 (6)	0	52 (8)	26 (4)	0.0007
MDR *Enterobacter* spp.—*n* (%)	44 (3)	0	44 (7)	–	–
Other *Enterobacterales*—*n* (%)	89 (7)	0	38 (6)	51 (7)	0.324
MDR other *Enterobacterales*—*n* (%)	13 (1)	0	13 (2)		
Non-fermenting Gram-negative bacilli—*n* (%)	73 (6)	0	49 (8)	24 (4)	0.001
MDR Non-fermenting Gram-negative bacilli—*n* (%)	29 (2)	0	29 (5)	–	–
*Pseudomonas aeruginosa*—*n* (%)	65 (5)	0	44 (7)	21 (3)	0.0013
MDR *Pseudomonas aeruginosa*—*n* (%)	24 (2)	0	24 (4)	–	–
Miscellaneous Gram-negative organisms—*n* (%)	6 (0.5)	0	1 (0.2)	5 (0.7)	0.220
Anaerobes—*n* (%)	116 (9)	0	48 (8)	68 (10)	0.145
*Bacteroides* spp.—*n* (%)	78 (6)	0	32 (5)	46 (7)	0.242
Other anaerobes—*n* (%)	38 (3)	0	16 (3)	22 (3)	0.512
Fungi—*n* (%)	157 (12)	0	73 (12)	84 (12)	0.697
*Candida albicans*—*n* (%)	94 (7)	0	36 (6)	58 (8)	0.054
*Non-albicans* Candida spp.—*n* (%)	54 (4)	0	33 (5)	21 (3)	0.052

The number of cultures sampled in each group was used as denominator for categorical variables

**Table 3:** Antibiotic susceptibilities expressed as proportions of Gram-negative bacilli without (no MDR) and with MDR profile

	AMC		TZP		IMP		CTX		CAZ		GEN		AMK		CIP		LVX	
	No MDR	MDR	No MDR	MDR	No MDR	MDR	No MDR	MDR	No MDR	MDR	No MDR	MDR	No MDR	MDR	No MDR	MDR	No MDR	MDR
*Enterobacterales* (*n *= 426)	68	5	99	38	100	97	99	55	100	64	99	77	99	91	100	86	98	71
*Escherichia coli* (*n *= 200)	82	7	100	60	100	98	100	85	100	88	100	81	99	99	100	96	97	73
*Klebsiella* spp. (*n *= 59)	85	5	95	21	100	94	100	37	100	39	100	39	100	74	100	63	100	56
*Enterobacter* spp. (*n *= 78)	–	–	100	5	100	97	93	5	100	10	96	79	100	79	100	65	97	70
Other *Enterobacterales* (*n *= 89)	75	0	100	23	100	100	100	31	100	40	100	69	99	85	100	86	100	83
Non-fermenting GNB (*n *= 73)	–	–	88	38	81	46	–	–	93	48	58	19	93	78	95	32	–	–
*Pseudomonas aeruginosa* (*n *= 65)	–	–	93	42	90	50	–	–	100	53	60	23	98	86	95	33	–	–

*AMC* Amoxicillin/clavulanic acid, *TZP* Piperacillin/tazobactam, *IMP* Imipenem/cilastatin, *CTX* Cefotaxime, *CAZ* Ceftazidime, *GEN* Gentamicin, *AMK* Amikacin, *CIP* Ciprofloxacin, *LVX* Levofloxacin

**Table 4:** Antibiotic susceptibilities expressed as proportions of Gram-positive cocci without (no MDR) and with MDR profile

	AMX		TZP		GEN		LVX		VAN		OXA	
	No MDR	MDR	No MDR	MDR	No MDR	MDR	No MDR	MDR	No MDR	MDR	No MDR	MDR
Enterococci (*n *= 254)	89	0	88	0	87	6	–	–	96	98	–	–
*Enterococcus faecalis* (*n *= 144)	100	–	100	–	82	–	–	–	100	–	–	–
*Enterococcus faecium* (*n *= 70)	38	0	31	0	95	7	–	–	100	97	–	–
Streptococci (*n *= 129)	98	–	83	–	98	–	–	–	100	–	–	–
Coagulase-negative Staphylococci (*n *= 82)	–	–	4	–	25	–	19	–	100	–	18	–
*Staphylococcus aureus* (*n *= 35)	100	–	100	–	100	88	100	20	100	100	100	0

*AMX* Amoxicillin, *TZP* Piperacillin/tazobactam, *GEN* Gentamicin, *LVX* Levofloxacin, *VAN* Vancomycin, *OXA* Oxacillin

**Table 5:** Antibiotic susceptibilities expressed as proportions of anaerobic bacteria

	AMX	AMC	TZP	IMP	MTR
*Clostridium* spp (*n *= 22)	86	100	100	100	100
*Bacteroides* spp (*n *= 78)	6	88	96	100	98
Other anaerobic bacteria (*n *= 16)	100	92	92	100	93

*AMX* Amoxicillin, *AMC* Amoxicillin/clavulanic acid, *TZP* Piperacillin/tazobactam, IMP Imipenem/cilastatin, *MTR* Metronidazole

Microbiological cultures identified 188/422 (45%) patients with MDRO and 234/422 (55%) patients free of MDRO. In these MDRO patients, a total of 243 (38%) MDROs were cultured among the 632 isolates (Table 2; Additional file 1: Tables S2 and S4), involving mostly GNB [201/632 (32% of the isolates)] and few GPC [42/632 (7%)]. A single MDRO was reported in 143/188 (76%) patients related to GNB in 122 patients (109 *Enterobacterales* and 13 non-fermenting GNB) and GPC in 21 patients (including 16 *E. faecium* and 4 MRSA). Two MDROs were cultured in 36/188 (19%) patients, including two GNB (22 patients) and mixed GNB and GPC (14 patients). Nine patients had mixed infections with 3 or 4 MDROs. Overall, MDR *Enterobacterales* were isolated in 150 patients, including ESBL-producing strains in 32 patients.

### Temporal changes in microbiological features

**Fig. 1:**
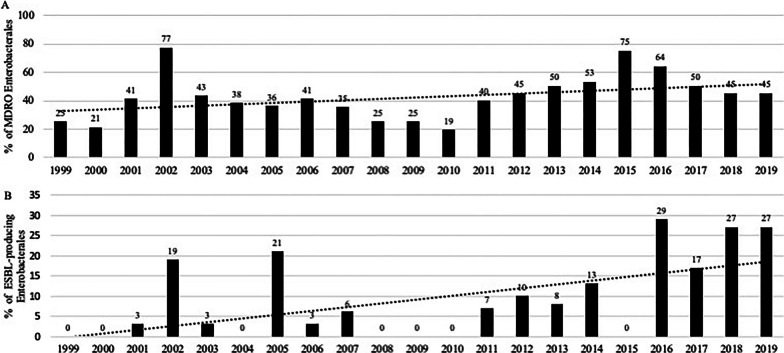
Annual proportions of MDR *Enterobacterales* (panel **A**) and ESBL-producing *Enterobacterales* (panel **B**). Results expressed as proportions in the family. *Footnote*: (panel **A**. Linear regression in dotted line *R*^2^ = 0.143; Cochran–Armitage test *p *= 0.0162) (panel **B**. Linear regression *R*^2^ = 0.320; Cochran–Armitage test *p *= 0.0013)

**Fig. 2:**
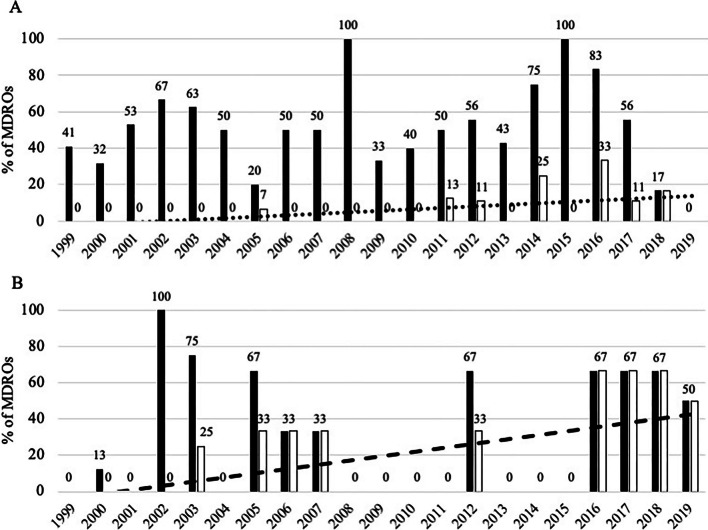
Annual proportions of MDR and ESBL-producing *Escherichia coli* (panel **A**) and *Klebsiella* spp (panel **B**). Results expressed as proportions in the family. *Footnote* (panel **A**. Linear regression in dotted line for ESBL-producing strains *R*^2^ = 0.283; Cochran–Armitage test *p *< 0.001 for ESBL-producing strains, respectively) (panel **B**. Linear regression in dotted line for ESBL-producing strains *R*^2^ = 0.306; Cochran–Armitage test *p *= 0.031 and *p *< 0.001 for MDR and ESBL-producing strains, respectively)

Overall, 18 [14–21] % (median, IQR) of the isolates were MDROs without any change in their yearly incidence (Cochran–Armitage test *p *= 0.09), leading to 43 [35–54] % of the patients harbouring MDROs (*p *= 0.19) (Additional file 1: Fig. S2). However, growing proportions of MDR *Enterobacterales* were observed over time (*p *= 0.016), including ESBL-producing *Enterobacterales* (*p *= 0.0013) (Fig. 1). Among MDR *Enterobacterales*, a trend towards increasing proportions of MDR *Klebsiella* spp was observed (*p *= 0.031). In addition, a significant increase in the proportions of ESBL-producing *E. coli* and *Klebsiella* spp. was reported (*p *< 0.001 in both cases) (Fig. 2). No MRSA was cultured from surgical samples between 2012 and 2019. Only one carbapenemase-producing *E. coli* (OXA-48) was observed during the study, while no vancomycin-resistant *E. faecalis* or *E. faecium* were reported. No other temporal changes were observed for any of the other MDROs (Additional file 1: Figs. S3 and S4).

**Fig. 3:**
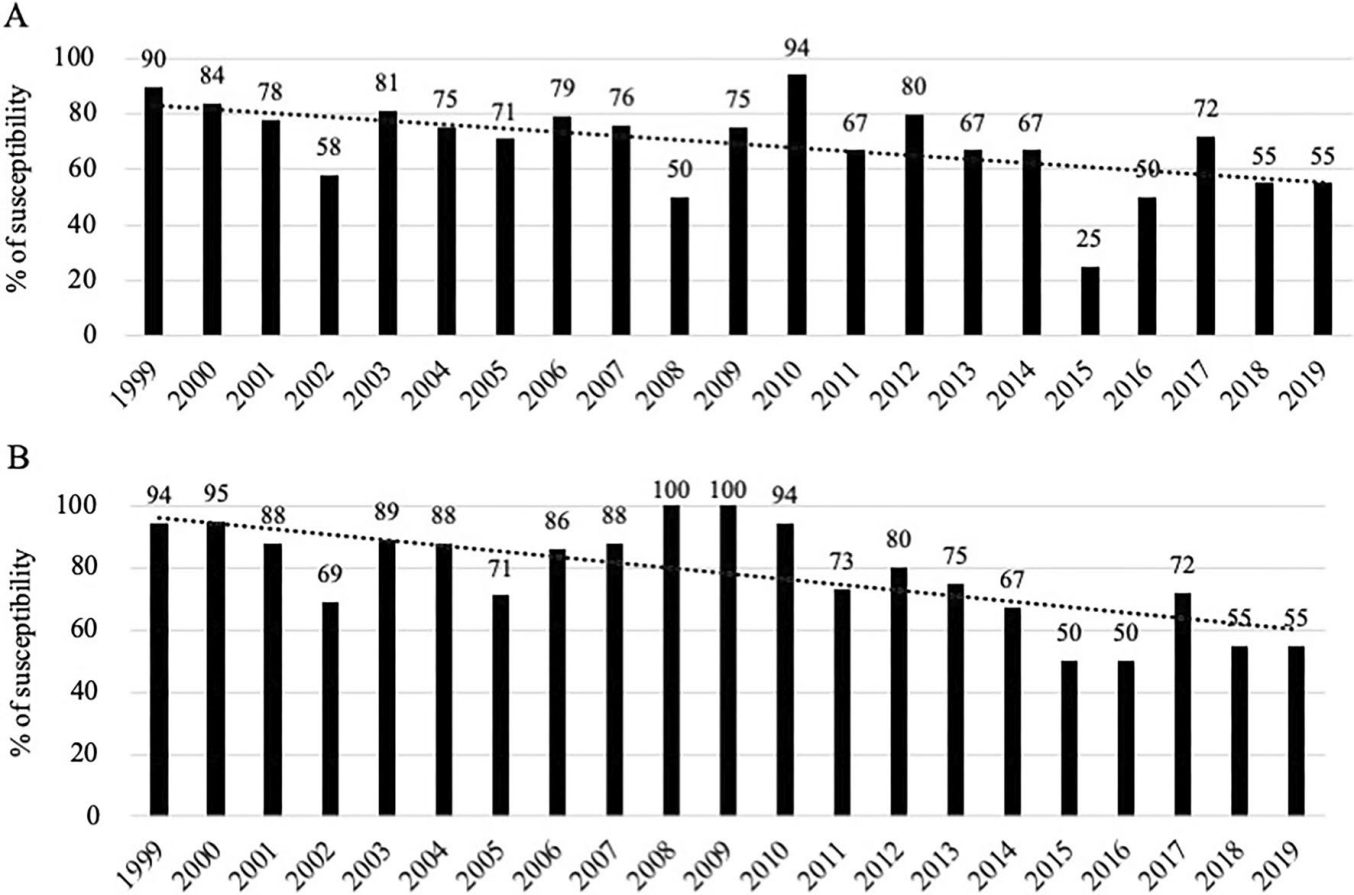
Annual susceptibility of *Enterobacterales* to piperacillin/tazobactam (panel **A**) and cefotaxime (panel **B**). Results expressed as proportions for the drug. *Footnote* (panel **A**. Linear regression in dotted line *R*^2^ = 0.298; Cochran–Armitage test *p *= 0.0005) (panel **B**. linear regression *R*^2^ = 0.480; Cochran–Armitage test *p *< 0.0001)

The only significant changes in the susceptibility profile of the isolates over the study period were a decreased susceptibility of *Enterobacterales* to piperacillin/tazobactam and cefotaxime (*p *= 0.0005 and *p *< 0.0001, respectively) (Fig. 3), while the susceptibility profile of other organisms remained unchanged (Additional file 1: Figs. S5 to S8).

### Empirical anti-infective therapy

Overall, 420/422 (99%) patients received EAT at the time of reoperation for POP. Two patients who did not receive any EAT were deemed to have inadequate EAT. The most frequent agents used for EAT are described in Table 6.

**Table 6:** Characteristics of the anti-infective therapy in the study population

Variables	Overall *N *= 422 patients	Missing value	MDRO group *N *= 188 patients	MDRO-free group *N *= 234 patients	*p* value
Empirical anti-infective therapy of POP*					
Monotherapy—*n* (%)	76 (18)	0	34 (18)	42 (18)	1.000
Carbapenem—*n* (%)	7 (2)	0	5 (3)	2 (1)	0.249
Piperacillin/tazobactam—*n* (%)	61 (15)	0	24 (13)	37 (16)	0.458
Two-drug regimen—*n* (%)	138 (33)	0	63 (34)	74 (32)	0.680
Three-drug regimen—*n* (%)	145 (35)	0	65 (35)	81 (35)	0.993
Four-drug regimen—*n* (%)	56 (13)	0	24 (13)	32 (14)	0.784
Carbapenem—*n* (%)	116 (28)	0	71 (38)	45 (19)	< 0.0001
Piperacillin/tazobactam—*n* (%)	246 (59)	0	97 (52)	149 (64)	0.0124
Aminoglycosides—*n* (%)	208 (50)	0	98 (52)	110 (47)	0.295
Vancomycin—*n* (%)	175 (42)	0	79 (42)	96 (41)	0.836
Antifungal therapy—*n* (%)	163 (39)	0	68 (36)	95 (41)	0.353
Adequacy of empirical antibiotic therapy*					
Overall adequacy—*n* (%)	305 (73)	0	115 (61)	190 (81)	0.0001
Adequate monotherapy—*n* (%)	44 (10)	0	10 (5)	34 (15)	0.002
Carbapenem—*n* (%)	3 (1)	0	2 (1)	1 (0.5)	0.587
Piperacillin/tazobactam—*n* (%)	39 (9)	0	7 (4)	32 (14)	0.0003
Adequate combination therapy—*n* (%)	261 (62)	0	105 (56)	156 (67)	0.026
Two-drug regimen—*n* (%)	93 (22)	0	34 (18)	59 (25)	0.097
Three-drug regimen—*n* (%)	114 (27)	0	50 (27)	64 (27)	0.912
Four-drug regimen—*n* (%)	50 (12)	0	20 (11)	30 (13)	0.546
Carbapenem part of the regimen—*n* (%)	91 (22)	0	55 (29)	36 (15)	0.0008
Piperacillin/tazobactam part of the regimen—*n* (%)	146 (35)	0	46 (24)	100 (43)	0.0001
Aminoglycosides part of the regimen—*n* (%)	169 (40)	0	72 (38)	97 (41)	0.549
Vancomycin part of the regimen—*n* (%)	153 (36)	0	64 (34)	89 (38)	0.416
Adequacy of EAT depending on the type of microorganisms*					
*Enterobacterales*—*n* (%)	224 (53)	0	103 (55)	121 (52)	0.556
MDR *Enterobacterales*—*n* (%)	95 (23)	0	95 (51)	*–*	*–*
*P. aeruginosa*—*n* (%)	40 (10)	0	22 (12)	18 (8)	0.182
MDR *P. aeruginosa*—*n* (%)	13 (3)	0	13 (7)	*–*	*–*
Enterococci—*n* (%)	153 (36)	0	66 (35)	87 (37)	0.684
MDR enterococci—*n* (%)	17 (4)	0	17 (9)	*–*	*–*
Coagulase-positive staphylococci—*n* (%)	26 (6)	0	8 (4)	18 (8)	0.159
MRSA—*n* (%)	5 (1)	0	5 (3)	*–*	*–*
*Documented anti-infective therapy#*					
Monotherapy—*n* (%)	135 (32)	0	97 (41)	38 (20)	< 0.0001
Two-drug regimen—*n* (%)	141 (33)	0	77 (33)	64 (34)	0.003
Three-drug regimen—*n* (%)	110 (26)	0	47 (20)	63 (34)	0.654
Four-drug regimen—*n* (%)	24 (6)	0	10 (4)	14 (7)	0.834
Carbapenem—*n* (%)	94 (22)	0	73 (39)	21 (9)	< 0.0001
Piperacillin/tazobactam—*n* (%)	124 (29)	0	50 (27)	74 (32)	0.259
Aminoglycosides—*n* (%)	58 (14)	0	35 (19)	23 (10)	0.009
Vancomycin—*n* (%)	121 (29)	0	72 (38)	49 (21)	< 0.0001
Antifungal therapy—*n* (%)	157 (37)	0	74 (39)	83 (35)	0.120
Duration of anti-infective therapy—days, median [IQR]	10 [7–14]	0	10 [7–14]	10 [7–14]	0.307

*Proportions of the overall population calculated on 420 patients receiving EAT

^#^Proportions of the overall population calculated on 422 patients receiving documented anti-infective therapy

Over the study period, the number of patients receiving imipenem/cilastatin and vancomycin remained unchanged (*p *= 0.073 and *p *= 0.544, respectively), while a significantly decreased number of piperacillin/tazobactam and aminoglycoside EAT was recorded (*p *= 0.0001 and *p *< 0.0001, respectively).

Overall, adequate EAT was achieved in 305/420 (73%) patients, with a significant decrease when MDROs were cultured (*p *= 0.0001) (Table 6). Adequacy was more frequently observed in combination therapies [261/344 (76%) vs. 44/76 (58%) monotherapies, respectively; *p *= 0.048]. The same benefit of combination therapy versus monotherapy was observed in the subgroup with MDRO [105/154 (68%) vs. 10/34 (29%), *p *< 0.0001] but not in the group free of MDRO [156/192 (81%) vs. 34/42 (81%), NS]. The adequacy of EAT remained stable over time (*p *= 0.460) (Additional file 1: Fig. S9).

### Best theoretical empirical antibiotic therapy

**Fig. 4:**
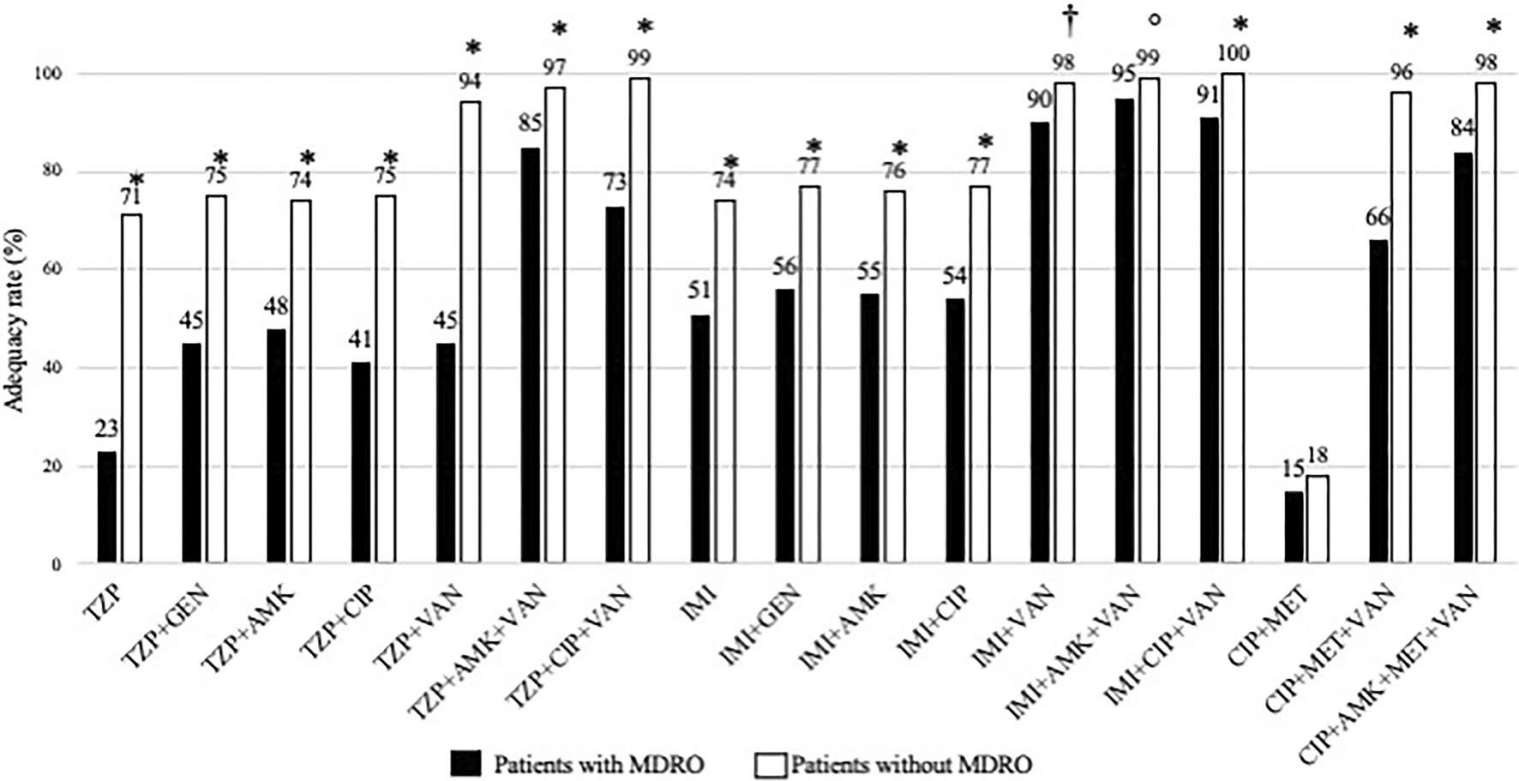
Theoretical adequacy rates of EAT expressed in proportions with various regimens for patients with/without MDRO during the 1999–2019 period. *p < 0.0001; †*p *< 0.01; °*p *< 0.05 versus MDROs with the same regimen. *Footnote* AMK: amikacin; CIP: ciprofloxacin; GEN: gentamicin; IMI: imipenem/cilastatin; MET: metronidazole; TZP: piperacillin/tazobactam; VAN: vancomycin

The theoretical adequacy of the 17 EAT therapeutic regimens is presented in Fig. 4. The highest rates of adequacy were reported for combination therapies including imipenem/cilastatin in both patients with and without MDRO (Fig. 4 and Additional file 1: Table S5 and Fig. S10). In patients with MDRO, combinations of imipenem/cilastatin + vancomycin + amikacin or ciprofloxacin demonstrated the highest adequacy rates, reaching 95% and 91% of the cases, respectively, and remained unchanged. In patients without MDRO, combinations of piperacillin/tazobactam + vancomycin + amikacin or ciprofloxacin reached similar levels of adequacy (97 and 99% respectively).

Analysis of the temporal trends in patients with MDROs showed decreased adequacy for piperacillin/tazobactam in monotherapy or combined with vancomycin and imipenem/cilastatin + vancomycin (*p *< 0.01 in all cases) (Additional file 1: Table S6 and Fig. S9). In the absence of MDRO, the adequacy of EAT with two or three drug regimens, including piperacillin/tazobactam or imipenem/cilastatin, remained stable over time (Additional file 1: Table S6).

## Discussion

This analysis of temporal trends for susceptibility of surgical specimens collected for POP indicates high and sustained proportions of MDROs involving 45% of the patients. Significantly increased proportions of MDR *Enterobacterales* were assessed, including ESBL-producing *Enterobacterales*. The adequacy of EAT was decreased for regimens involving both piperacillin/tazobactam monotherapy and combination, while the adequacy rate remained stable for carbapenems in combination with aminoglycosides or fluroquinolones. To our knowledge, this is the first report analysing yearly MDRO rates of POP in one hospital using a consistent definition of resistance.

Several definitions of MDRO have been proposed, some of which are limited to GNB [23, 24]. We used the criteria proposed by the ECDC, accounting for GNB and GPC [21]. The studies comparing MDRO definitions reported high variability of the resistance rates, with the ECDC definitions being associated with the highest rates of resistance [24]. The ECDC definitions have been criticized as they encompass resistance to many antibiotic families, including those rarely used in IAIs such as monobactams, non-beta-lactam cell wall synthesis inhibitors, protein synthesis inhibitors or antifolate agents [23, 24]. This issue led us to broaden our analysis and to assess the changes in adequacy of EAT and the best theoretical treatments over the study period.

Several authors have described the emergence of MDROs in IAIs [13, 25, 26]. Some did not provide any definition of MDRO [27]. More recently, MRSA has been clearly defined [13, 26, 28]. The definition of multidrug resistance for enterococci is sometimes limited to glycopeptide/vancomycin resistance [13]. More variability has been reported in the definitions of GNB. Seguin et al. assessed ESBL- or high-level-producing cephalosporinases *Enterobacterales*, while their definition of *Pseudomonas aeruginosa* resistance was focused on ticarcillin, ceftazidime, imipenem or ciprofloxacin resistance, or ESBL-producing strains. In the AbSeS trial, antimicrobial resistance for GNB was defined as ESBL-producing strains, carbapenem resistance, or fluoroquinolone resistance [13]. The authors also identified “difficult-to-treat” resistance for GNB as combination resistance to all tested carbapenem, other beta-lactam, and fluoroquinolone agents. These features dramatically limit the comparisons between the cohorts.

In our population, higher proportions of MDROs were observed compared to previous reports. Seguin et al. [26] reported 17% of patients with MDROs, involving 90% of *Enterobacterales*, mainly related to high-level cephalosporinases. When considering the AbSeS trial, our rates of MDRO are close to the proportions observed in Southern Europe (27.3–28.5% of patients with MDROs) rather than those reported in Western Europe (9.8–15.4% of the patients), but we only evidenced limited proportions of MDR-GPC.

In terms of the timeline, we observed progressively increased proportions of ESBL-producing strains. This has been previously reported in other infections, including urinary tract infections, but not during POP [29]. Furthermore, the emergence of carbapenem-resistant *Enterobacterales* has remained marginal over recent years in France [30]. Across Europe, carbapenem-resistant GNB are quite rarely observed, except in Southern and South‒East countries [13]. Interestingly, NF-GNB were only reported in a limited number of cases, consistent with previous reports, with low proportions of resistance and no significant change over time [13, 14, 26, 27]. These observations confirm that these organisms are not usual targets for EAT. Enterococci are another source of concern despite low proportions of vancomycin-resistant MDROs. However, the high rate of penicillin-nonsusceptible enterococci leads to the consideration of a specific anti-GPC EAT. The same conclusions were drawn in Germany from a cohort of 422 cases of POP with high proportions of *E. faecium*, all resistant to penicillins [25].

Our observations confirm the challenging issue of EAT and the need for combination therapy. The progressive decreased susceptibility of piperacillin/tazobactam in patients with POP over the study period is not a surprise in light of emerging resistance reported worldwide [13]. To date, in Western Europe, this issue has not been described in IAIs. In our data, even in combination with glycopeptides, piperacillin/tazobactam reached less than 70% adequacy in patients harbouring MDROs. Only a limited number of combinations reached more than 90% adequacy against all patients. The good news is the remaining capacity of some combinations for targeting all the organisms, but unfortunately the need for carbapenem remains crucial in many instances.

The optimization of antibiotic therapy is a key issue. The routine use of broad-spectrum therapies, including carbapenems, only minimally improved the adequacy rate in infections involving MDROs, still ranging between 30 and 60% of the cases [26, 31, 32]. These observations are a plea for the use of genomic analysis techniques for rapid pathogen identification and assessment of resistance genes within a few hours. These new microbiological diagnostic methods make it possible to move to an oriented prescription, reducing the delay for selection of the most accurate therapeutic strategies. Promising results have been obtained in several sites of infection. Only a few data are available in IAIs thus far [33].

The second point of importance for improving EAT is to consider the evolution of resistance patterns. Longitudinal evaluation through databases could be of value for the detection of the emergence of resistance in specific populations. While national or regional databases are not accurate enough for improving EAT of healthcare-associated infections, computer-assisted prescriptions at the hospital level have already demonstrated their ability in selecting the most adequate antibiotic therapy, even in critically ill patients [34, 35].

The main limitation of our analysis is the retrospective, monocentric nature of the study. Any extrapolation to another institution must be considered cautiously. However, our results are consistent with previous publications [25]. For the purpose of this registry, we collected only limited clinical information during the ICU stay. Since our purpose was a pragmatic analysis of our cases, we did not investigate in detail the mechanisms of resistance, which limits the relevance of our observations. Over the last few decades, some modifications in minimal inhibitory concentration breakpoints have been implemented that might also influence our results, even if they remain marginal [20]. Our assumptions on the efficacy of EAT are strictly a theoretical approach not validated in clinical practice. In addition, these hypotheses did not consider potential pharmacokinetic changes related to sepsis [36]. Finally, these observations are not applicable to persistent postoperative peritonitis or recurrent infections, in which conditions higher rates of resistance have been reported [37]. The principal strengths of this study are that these data are derived from one institution, the screening definition for POP has remained unchanged, and the same medical, surgical, and microbiology teams have been responsible for data collection throughout the study. Thus, we assume that our observations could have some relevance for clinicians and could be an incentive for close follow-up of their local epidemiology.

In conclusion, this longitudinal evaluation of resistance patterns suggests a high incidence of MDRO among the surgical samples of patients with POP. In addition, a progressive increase in the rate of MDR *Enterobacterales* was observed with a specific reference for ESBL-producing strains, while the other families remain stable. Only combination therapies provide a high probability of adequate EAT. Piperacillin/tazobactam is no longer a drug of choice for EAT in POP in infections involving MDRO. The optimization of EAT should be based on local analysis of resistance patterns to select the regimens providing the highest adequacy rates. A large spread of computer-assisted prescriptions based on local databases combined with rapid diagnostic tests will help in selecting the most appropriate EAT.

### Supplementary Information

**Additional file 1. Supplementary text. **Management of microbiological samples. **Table S1**. STROBE Statement. **Table S2**. Microbiologically documented surgical samples expressed as numbers or proportions per patient. **Table S3**. Temporal changes in the proportions of cultured organisms in the study population assessed with the Cochrane Armitage test. **Table S4**. Detailed microbiological resistance profile according to the recovered pathogens. **Table S5**. Crude rates of theoretical adequate EAT achieved with various regimens assessed in patients with/without MDROs between 1999 and 2019. **Table S6**. Temporal changes in the rate of theoretical adequate EAT for various regimens assessed with Cochrane Armitage test. **Figure S1**. Study flowchart. **Figure S2**. Annual proportion of MDROs expressed per total number of microorganisms (**A**) and per patient (**B**). **Figure S3**. Annual proportion of MDR and ESBL-producing Enterobacter spp. (**A**) and MDR and ESBL-producing other Enterobacterales (**B**) expressed in proportion of isolates in the family. MDR strains in closed boxes and ESBL-producing strains in open boxes. **Figure S4**. Annual proportion of MDR nonfermenting Gram-negative bacilli (**A**), MDR enterococci (**B**), and MDR Staphylococcus aureus (**C**) expressed in proportion of isolates in the family. **Figure S5**. Annual susceptibility of Enterobacterales to imipenem/cilastatin (**A**), amikacin (**B**) and levofloxacine/ciprofloxacin (**C**). **Figure S6**. Annual susceptibility of nonfermenting Gram-negative bacilli to piperacillin/tazobactam (**A**), ceftazidime (**B**), imipenem/cilastatin (**C**), amikacin (**D**) and ciprofloxacin (**E**). **Figure S7**. Annual susceptibility of enterococci to amoxicillin (**A**), gentamicin (**B**), and vancomycin (**C**). **Figure S8**. Annual susceptibility of Staphylococcus aureus to oxacillin (**A**) gentamicin (**B**) ofloxacin/levofloxacin (**C**) and vancomycin (**D**). **Figure S9**. Annual adequacy of EAT in the study population (**A**), in the group of patients with MDRO (**B**), and in the group of patients without MDRO (**C**). **Figure S10**. Annual theoretical rate of adequacy of piperacillin/tazobactam (**A**), piperacillin/tazobactam + vancomycin (**B**), and imipenem/cilastatin + vancomycin (**C**) in patients with (red boxes) and without MDROs (open boxes).

## Data Availability

The datasets generated during and/or analysed during the current study are available from the corresponding author on reasonable request.

## Abbreviations

EAT: Empirical antibiotic therapy
ECDC: European Centre for Disease Prevention and Control
ESBL: Extended-spectrum beta-lactamase
EUCAST: European Committee on Antimicrobial Susceptibility Testing
GNB: Gram-negative bacilli
GPC: Gram-positive cocci
IAIs: Intraabdominal infections
ICU: Intensive care unit
IQRs: Interquartile ranges
MDRO: Multidrug-resistant organism
MRSA: Methicillin-resistant *Staphylococcus aureus*
POP: Postoperative peritonitis
SAPS: Simplified acute physiologic score
SOFA: Sequential organ failure assessment
