# Supplementary material for: Microbiological profile of patients treated for postoperative peritonitis: temporal trends 1999–2019
Source: World J Emerg Surg. 2023 Dec 19;18:58. doi: 10.1186/s13017-023-00528-1 (PMC10729506; doi:10.1186/s13017-023-00528-1)
Supplement: Supplementary file 1 — Additional file 1. Supplementary text. Management of microbiological samples. Table S1. STROBE Statement. Table S2. Microbiologically documented surgical samples expressed as numbers or proportions per patient. Table S3. Temporal changes in the proportions of cultured organisms in the study population assessed with the Cochrane Armitage test. Table S4. Detailed microbiological resistance profile according to the recovered pathogens. Table S5. Crude rates of theoretical adequate EAT achieved with various regimens assessed in patients with/without MDROs between 1999 and 2019. Table S6. Temporal changes in the rate of theoretical adequate EAT for various regimens assessed with Cochrane Armitage test. Figure S1. Study flowchart. Figure S2. Annual proportion of MDROs expressed per total number of microorganisms (A) and per patient (B). Figure S3. Annual proportion of MDR and ESBL-producing Enterobacter spp. (A) and MDR and ESBL-producing other Enterobacterales (B) expressed in proportion of isolates in the family. MDR strains in closed boxes and ESBL-producing strains in open boxes. Figure S4. Annual proportion of MDR nonfermenting Gram-negative bacilli (A), MDR enterococci (B), and MDR Staphylococcus aureus (C) expressed in proportion of isolates in the family. Figure S5. Annual susceptibility of Enterobacterales to imipenem/cilastatin (A), amikacin (B) and levofloxacine/ciprofloxacin (C). Figure S6. Annual susceptibility of nonfermenting Gram-negative bacilli to piperacillin/tazobactam (A), ceftazidime (B), imipenem/cilastatin (C), amikacin (D) and ciprofloxacin (E). Figure S7. Annual susceptibility of enterococci to amoxicillin (A), gentamicin (B), and vancomycin (C). Figure S8. Annual susceptibility of Staphylococcus aureus to oxacillin (A) gentamicin (B) ofloxacin/levofloxacin (C) and vancomycin (D). Figure S9. Annual adequacy of EAT in the study population (A), in the group of patients with MDRO (B), and in the group of patients without MDRO (C). Figure S10. Annual [file 13017_2023_528_MOESM1_ESM.docx]

Supplementary material

[Supplementary text. - Management of microbiological samples 2](#_Toc152424947)

[Supplementary Table S1. STROBE Statement 3](#_Toc152424948)

[Table S2. Microbiologically documented surgical samples expressed as numbers or proportions per patient. 5](#_Toc152424949)

[Table S3. Temporal changes in the proportions of cultured organisms in the study population assessed with the Cochrane Armitage test. 6](#_Toc152424950)

[Table S4. Detailed microbiological resistance profile according to the recovered pathogens 7](#_Toc152424951)

[Table S5. Crude rates of theoretical adequate EAT achieved with various regimens assessed in patients with/without MDROs between 1999 and 2019. 8](#_Toc152424952)

[Table S6. Temporal changes in the rate of theoretical adequate EAT for various regimens assessed with Cochrane Armitage test. 9](#_Toc152424953)

[Supplementary Figure S1. Study flowchart. 10](#_Toc152424954)

[Figure S2. Annual proportion of MDROs expressed per total number of microorganisms (A) and per patient (B). 11](#_Toc152424955)

[Figure S3. Annual proportion of MDR and ESBL-producing *Enterobacter* spp. (A) and MDR and ESBL-producing other *Enterobacterales* (B) expressed in proportion of isolates in the family. MDR strains in closed boxes and ESBL-producing strains in open boxes. 12](#_Toc152424956)

[Figure S4. Annual proportion of MDR nonfermenting Gram-negative bacilli (A), MDR enterococci (B), and MDR *Staphylococcus aureus* (C) expressed in proportion of isolates in the family. 13](#_Toc152424957)

[Figure S5. Annual susceptibility of *Enterobacterales* to imipenem/cilastatin (A), amikacin (B) and levofloxacine/ciprofloxacin (C). 14](#_Toc152424958)

[Figure S6. Annual susceptibility of nonfermenting Gram-negative bacilli to piperacillin/tazobactam (A), ceftazidime (B), imipenem/cilastatin (C), amikacin (D) and ciprofloxacin (E). 15](#_Toc152424959)

[Figure S7. Annual susceptibility of enterococci to amoxicillin (A), gentamicin (B), and vancomycin (C). 16](#_Toc152424960)

[Figure S8. Annual susceptibility of *Staphylococcus aureus* to oxacillin (A) gentamicin (B) ofloxacin/levofloxacin (C) and vancomycin (D). 17](#_Toc152424961)

[Figure S9. Annual adequacy of EAT in the study population (A), in the group of patients with MDRO (B), and in the group of patients without MDRO (C). 18](#_Toc152424962)

[Figure S10. Annual theoretical rate of adequacy of piperacillin/tazobactam (A), piperacillin/tazobactam + vancomycin (B), and imipenem/cilastatin + vancomycin (C) in patients with (red boxes) and without MDROs (open boxes). 19](#_Toc152424963)

# Supplementary text. - Management of microbiological samples

Peritoneal samples collected during reoperation were immediately sent to the microbiology laboratory. Samples were processed according to standard laboratory methods. After Gram staining for direct examination, samples were inoculated onto routine agar plates, which included trypticase soy agar supplemented with 5% horse blood, Columbia sheep blood agar containing nalidixic acid and colistin, chocolate agar supplemented with PolyVitex for isolation of fastidious bacteria, Drigalski agar for isolation of *Enterobacterales* and certain non-fermenters GNB, Candida agar for isolation of yeasts and Brain Heart Infusion broth. The plates and broth were incubated for 48 hours at 35°C±2 under aerobic and anaerobic conditions.

# Supplementary Table S1. STROBE Statement

|  | Item No | Recommendation | Page No |
| --- | --- | --- | --- |
| **Title and abstract** | 1 | (*a*) Indicate the study’s design with a commonly used term in the title or the abstract | 1 |
|  |  | (*b*) Provide in the abstract an informative and balanced summary of what was done and what was found | 2, lines 20-25 |
| Introduction | | | |
| Background/rationale | 2 | Explain the scientific background and rationale for the investigation being reported | 6, lines 70-83 |
| Objectives | 3 | State specific objectives, including any prespecified hypotheses | 6, lines 83-87 |
| Methods | | | |
| Study design | 4 | Present key elements of study design early in the paper | 7, lines 90-99 |
| Setting | 5 | Describe the setting, locations, and relevant dates, including periods of recruitment, exposure, follow-up, and data collection | 7-8 |
| Participants | 6 | (*a*) Give the eligibility criteria, and the sources and methods of case ascertainment and control selection. Give the rationale for the choice of cases and controls | 7, lines 95-9 |
|  |  | (*b*) For matched studies, give matching criteria and the number of controls per case | NA |
| Variables | 7 | Clearly define all outcomes, exposures, predictors, potential confounders, and effect modifiers. Give diagnostic criteria, if applicable | 7-9 |
| Data sources/ measurement | 8 | For each variable of interest, give sources of data and details of methods of assessment (measurement). Describe comparability of assessment methods if there is more than one group | 7-9 |
| Bias | 9 | Describe any efforts to address potential sources of bias | 7-9 |
| Study size | 10 | Explain how the study size was arrived at | 7, lines 91-3 |
| Quantitative variables | 11 | Explain how quantitative variables were handled in the analyses. If applicable, describe which groupings were chosen and why | 9-10 |
| Statistical methods | 12 | (*a*) Describe all statistical methods, including those used to control for confounding | 9-10 |
|  |  | (*b*) Describe any methods used to examine subgroups and interactions | 9-10 |
|  |  | (*c*) Explain how missing data were addressed | 10, lines 71-2 |
|  |  | (*d*) If applicable, explain how matching of cases and controls was addressed | NA |
|  |  | (*e*) Describe any sensitivity analyses | NA |
| Results | | | |
| Participants | 13 | (a) Report numbers of individuals at each stage of study—eg numbers potentially eligible, examined for eligibility, confirmed eligible, included in the study, completing follow-up, and analysed | 11 and Supplementary Figure S1 |
|  |  | (b) Give reasons for non-participation at each stage | Supplementary Figure S1 |
|  |  | (c) Consider use of a flow diagram | Supplementary Figure S1 |
| Descriptive data | 14 | (a) Give characteristics of study participants (eg demographic, clinical, social) and information on exposures and potential confounders | 11 + Table 1 |
|  |  | (b) Indicate number of participants with missing data for each variable of interest | 11-6  Tables1,2,4 |
| Outcome data | 15 | Report numbers in each exposure category, or summary measures of exposure | 11-8  Tables 1-4 and supplementary tables S2-S4 |

| Main results | | 16 | (*a*) Give unadjusted estimates and, if applicable, confounder-adjusted estimates and their precision (eg, 95% confidence interval). Make clear which confounders were adjusted for and why they were included | 11-8 |
| --- | --- | --- | --- | --- |
|  |  |  | (*b*) Report category boundaries when continuous variables were categorized | 11-8 |
|  |  |  | (*c*) If relevant, consider translating estimates of relative risk into absolute risk for a meaningful time period | NA |
| Other analyses | 17 | Report other analyses done—eg analyses of subgroups and interactions, and sensitivity analyses | | Tables 1-4, and supplementary Tables S2-S7  Figures 1-2 and supplementary Figures S2-10 |
| Discussion | | | | |
| Key results | 18 | Summarise key results with reference to study objectives | | 19, lines 266-73 |
| Limitations | 19 | Discuss limitations of the study, taking into account sources of potential bias or imprecision. Discuss both direction and magnitude of any potential bias | | 22, lines 338-54 |
| Interpretation | 20 | Give a cautious overall interpretation of results considering objectives, limitations, multiplicity of analyses, results from similar studies, and other relevant evidence | | 19-21 |
| Generalisability | 21 | Discuss the generalisability (external validity) of the study results | | 19, lines 347-9 |
| Other information | | | | |
| Funding | 22 | Give the source of funding and the role of the funders for the present study and, if applicable, for the original study on which the present article is based | | 24, line 380 |

# Table S2. Microbiologically documented surgical samples expressed as numbers or proportions per patient.

| Organisms | **Overall**  N=422 patients | **Missing**  **value** | **MDRO group**  N=188 patients | **MDRO-free group**  N=234 patients | ***p* value** |
| --- | --- | --- | --- | --- | --- |
| Monomicrobial peritoneal samples – n (%) ^a^ | 51 (12) | 0 | 18 (10) | 33 (14) | 0.161 |
| Gram-positive aerobic cocci – n (%) | 340 (81) | 0 | 143 (76) | 197 (84) | 0.036 |
| MDR Gram-positive aerobic cocci – n (%) | 40 (9) | 0 | 40 (21) | - | - |
| *Streptococcus* spp. – n (%) | 104 (25) | 0 | 30 (16) | 74 (32) | 0.0002 |
| *Enterococcus* spp. – n (%) | 216 (51) | 0 | 108 (57) | 108 (46) | 0.021 |
| MDR enterococci – n (%) | 31 (7) | 0 | 31 (16) | - | - |
| *Enterococcus faecalis* – n (%) | 138 (33) | 0 | 61 (32) | 77 (33) | 0.920 |
| *Enterococcus faecium* – n (%) | 70 (17) | 0 | 47 (25) | 23 (10) | <0.0001 |
| MDR *Enterococcus faecium* – n (%) | 30 (7) | 0 | 30 (16) | - | - |
| Other enterococci – n (%) | 41 (10) | 0 | 21 (11) | 20 (9) | 0.365 |
| *Staphylococcus aureus* – n (%) | 35 (8) | 0 | 15 (8) | 20 (9) | 0.833 |
| MDR *Staphylococcus aureus* – n (%) | 11 (3) | 0 | 11 (6) | - | - |
| Coagulase-negative staphylococci – n (%) | 80 (19) | 0 | 34 (18) | 46 (20) | 0.682 |
| Miscellaneous Gram-positive cocci – n (%) | 7 (2) | 0 | - | 7 (3) | - |
| Gram-negative aerobic bacilli – n (%) | 329 (78) | 0 | 182 (97) | 147 (63) | <0.0001 |
| MDR Gram-negative aerobic bacilli – n (%) | 167 (40) | 0 | 167 (89) | - | - |
| *Enterobacterales* – n (%) | 304 (72) | 0 | 168 (89) | 136 (58) | <0.0001 |
| MDR *Enterobacterales* – n (%) | 150 (36) | 0 | 150 (80) | - | - |
| *Escherichia coli* – n (%) | 190 (45) | 0 | 110 (59) | 80 (34) | <0.0001 |
| MDR *Escherichia coli* – n (%) | 93 (22) | 0 | 93 (49) | - | - |
| *Klebsiella* spp. – n (%) | 59 (14) | 0 | 29 (15) | 30 (13) | 0.443 |
| MDR *Klebsiella* spp. – n (%) | 19 (5) | 0 | 19 (10) | - | - |
| *Enterobacter* spp. – n (%) | 75 (18) | 0 | 49 (26) | 26 (11) | <0.0001 |
| MDR *Enterobacter* spp. – n (%) | 41 (10) | 0 | 41 (22) | - | - |
| Other *Enterobacterales* – n (%) | 81 (19) | 0 | 37 (20) | 44 (19) | 0.820 |
| MDR other *Enterobacterales* – n (%) | 13 (3) | 0 | 13 (7) | - | - |
| Non-fermenting Gram-negative bacilli – n (%) | 71 (17) | 0 | 47 (25) | 24 (10) | <0.0001 |
| MDR Non-fermenting Gram-negative bacilli – n (%) | 29 (7) | 0 | 29 (15) | - | - |
| *Pseudomonas aeruginosa* – n (%) | 63 (15) | 0 | 42 (22) | 21 (9) | <0.0001 |
| MDR *Pseudomonas aeruginosa* – n (%) | 24 (6) | 0 | 24 (13) | - | - |
| Miscellaneous Gram-negative bacilli – n (%) | 82 (19) | 0 | 36 (19) | 46 (20) | 0.895 |
| Anaerobes – n (%) | 94 (22) | 0 | 39 (21) | 55 (24) | 0.498 |
| *Bacteroides* spp. – n (%) | 72 (17) | 0 | 30 (16) | 42 (18) | 0.588 |
| Other anaerobes – n (%) | 37 (9) | 0 | 16 (9) | 21 (9) | 0.867 |
| Fungi – n (%) | 150 (36) | 0 | 72 (38) | 78 (33) | 0.289 |
| *Candida albicans* – n (%) | 98 (23) | 0 | 40 (21) | 58 (25) | 0.396 |
| *Non-albicans* Candida spp. – n (%) | 54 (13) | 0 | 33 (18) | 21 (9) | 0.0087 |

Results are expressed as number of episodes and proportions of microorganisms per patient.

The number of patients with cultured samples was used as denominator.

# Table S3. Temporal changes in the proportions of cultured organisms in the study population assessed with the Cochrane Armitage test.

| Organisms | *p* value |
| --- | --- |
| Monomicrobial peritoneal sample | 0.184 |
| Gram-positive aerobic cocci | 0.364 |
| *Streptococcus* spp. | 0.536 |
| *Enterococcus* spp. | 0.171 |
| *Staphyloccus aureu*s | 0.136 |
| Gram-negative aerobic bacilli | 0.079 |
| *Enterobacterales* | 0.210 |
| Non-fermenting Gram-negative bacilli | 0.954 |
| *Pseudomonas aeruginosa* | 0.819 |

# Table S4. Detailed microbiological resistance profile according to the recovered pathogens

| Resistance profile | Type of pathogens |
| --- | --- |
| Gram-negative aerobes |  |
| *Enterobacterales* |  |
| ESBL producing *Enterobacterales* (n=34) | *E coli* (n=8) ; *Klebsiella* spp (n=12) ; *Enterobacter* spp (n=13) ; Other *Enterobacterales* (n=1) |
| Non ESBL-producing *Enterobacterales* resistant to third generation cephalosporin- (n=45) | MDR *E. coli* (n=6); MDR *Enterobacter* spp (n=29) and not-MDR *Enterobacter* spp strains (n=2) ; Other MDR *Enterobacterales* (n=9) (*Morganella morganii* (n=4), *Serratia* spp (n=1), *Citrobacter* spp (n=1), *Proteus* spp (n=1), *Hafnia alvei* (n=1)) |
| Imipenem-resistant *Enterobacterales* (n=3) | OXA-48-producing *E. coli* (n=1) ; MDR *K. pneumoniae* (n=1) and MDR *E. aerogenes* (n=1) (no mechanistic analysis for these two strains) |
| Non-fermenting Gram-negative bacilli |  |
| Ceftazidime-resistant NF-GNB (n=13) | MDR *Pseudomonas aeruginosa* (n= 8) ; MDR *Acinetobacter baumannii* (n=3) ; not-MDR *Stenotrophomonas maltophilia* (n=2) |
| Imipenem-resistant NF-GNB (n=20) | MDR *P. aeruginosa* (n= 11) ; MDR *Acinetobacter baumannii* (n=3) ; *P. aeruginosa* (n=3) and *S. maltophilia* (n=3) not MDR ; |
| Gram-positive aerobes |  |
| Amoxicillin-resistant Enterococci (n=56) | MDR *E. faecium* (n=31) and not-MDR *E. faecium* (n=24); MDR *E durans* (n=1) |
| Vancomycin-resistant Enterococci (n=9) | MDR *E. faecium* VAN-R (n=1), *E. casseliflavus* (n=5) and *E. gallinarum* (n=3) not MDR strains |
| *Staphylococcus aureus* | MDR Methicillin-resistant *S. aureus* (n=11) |

# Table S5. Crude rates of theoretical adequate EAT achieved with various regimens assessed in patients with/without MDROs between 1999 and 2019.

| Empirical regimens | Study population  (n=422 patients) | Patients with MDRO  (n=188 patients) | Patients without MDRO  (n=234 patients) | P value |
| --- | --- | --- | --- | --- |
| TZP, – n (%) | 211 (50) | 44 (23) | 167 (71) | <0.0001 |
| TZP+GEN, – n (%) | 260 (62) | 84 (45) | 176 (75) | <0.0001 |
| TZP+AMK, – n (%) | 264 (63) | 90 (48) | 174 (74) | <0.0001 |
| TZP+CIP, – n (%) | 252 (60) | 77 (41) | 175 (75) | <0.0001 |
| TZP+VAN, – n (%) | 304 (72) | 85 (45) | 219 (94) | <0.0001 |
| TZP+AMK+VAN, – n (%) | 337 (80) | 160 (85) | 227 (97) | <0.0001 |
| TZP+CIP+VAN, – n (%) | 370 (88) | 138 (73) | 232 (99) | <0.0001 |
| IMI, – n (%) | 269 (64) | 95 (51) | 174 (74) | <0.0001 |
| IMI+GEN, – n (%) | 285 (68) | 105 (56) | 180 (77) | <0.0001 |
| IMI+AMK, – n (%) | 281 (67) | 103 (55) | 178 (76) | <0.0001 |
| IMI+CIP, – n (%) | 280 (66) | 101 (54) | 179 (77) | <0.0001 |
| IMI+VAN, – n (%) | 399 (95) | 169 (90) | 230 (98) | 0.0002 |
| IMI+AMK+VAN, – n (%) | 409 (97) | 178 (95) | 231 (99) | 0.02 |
| IMI+CIP+VAN, – n (%) | 405 (96) | 171 (91) | 234 (100) | <0.0001 |
| CIP+MET, – n (%) | 69 (16) | 28 (15) | 41 (18) | 0.466 |
| CIP+MET+VAN, – n (%) | 348 (82) | 124 (66) | 224 (96) | <0.0001 |
| CIP+AMK+MET+VAN, – n (%) | 388 (92) | 158 (84) | 230 (98) | <0.0001 |

P value: patients with MDRO versus patients without MDRO with the same regimen.

AMK: amikacin; CIP: ciprofloxacin; GEN: gentamicin; IMI: imipenem/cilastatin; MET: metronidazole; TZP: piperacillin/tazobactam; VAN: vancomycin.

# Table S6. Temporal changes in the rate of theoretical adequate EAT for various regimens assessed with Cochrane Armitage test.

| Empirical antibiotic regimens | Study population  (n=422 patients) | Patients with MDRO  (n=188 patients) | Patients without MDRO  (n=234  patients) |
| --- | --- | --- | --- |
| TZP, – p value | 0.004 | 0.004 | 0.027 |
| TZP+GEN, – p value | 0.105 | 0.146 | 0.243 |
| TZP+AMK, – p value | 0.323 | 0.945 | 0.087 |
| TZP+CIP, – p value | 0.863 | 0.346 | 0.059 |
| TZP+VAN, – p value | 0.001 | 0.001 | 0.251 |
| TZP+AMK+VAN, – p value | 0.876 | 0.906 | 0.443 |
| TZP+CIP+VAN, – p value | 0.229 | 0.070 | 0.315 |
| IMI, – p value | 0.142 | 0.856 | 0.033 |
| IMI+GEN, – p value | 0.588 | 0.572 | 0.124 |
| IMI+AMK, – p value | 0.278 | 0.861 | 0.055 |
| IMI+CIP, – p value | 0.42 | 0.640 | 0.066 |
| IMI+VAN, – p value | 0.003 | 0.013 | 0.050 |
| IMI+AMK+VAN, – p value | 0.072 | 0.257 | 0.071 |
| IMI+CIP+VAN, – p value | 0.186 | 0.135 | 0.999 |
| CIP+MET, – p value | 0.084 | 0.880 | 0.033 |
| CIP+MET+VAN, – p value | 0.121 | 0.092 | 0.372 |
| CIP+AMK+MET+VAN – p value | 0.832 | 0.772 | 0.100 |

P value: patients with MDRO versus patients without MDRO with the same regimen.

AMK: amikacin; CIP: ciprofloxacin; GEN: gentamicin; IMI: imipenem/cilastatin; MET: metronidazole; TZP: piperacillin/tazobactam; VAN: vancomycin.

# Supplementary Figure S1. Study flowchart.

# Figure S2. Annual proportion of MDROs expressed per total number of microorganisms (A) and per patient (B).

# Figure S3. Annual proportion of MDR and ESBL-producing *Enterobacter* spp. (A) and MDR and ESBL-producing other *Enterobacterales* (B) expressed in proportion of isolates in the family. MDR strains in closed boxes and ESBL-producing strains in open boxes.

Cochran-Armitage test p=0.063 and p=0.267 for MDR strains in each family, respectively. Cochran-Armitage test p=0.708 and p=0.495 for ESBL-producing strains in each family, respectively.

# Figure S4. Annual proportion of MDR nonfermenting Gram-negative bacilli (A), MDR enterococci (B), and MDR *Staphylococcus aureus* (C) expressed in proportion of isolates in the family.

Cochran-Armitage test p=0.726, p=0.425 and p=0.209, for Gram-negative bacilli, enterococci and *Staphylococcus aureus*, respectively.

# Figure S5. Annual susceptibility of *Enterobacterales* to imipenem/cilastatin (A), amikacin (B) and levofloxacine/ciprofloxacin (C).

Cochran-Armitage test p=0.0136, p=0.841, and p=0.288, for imipenem/cilastatin, amikacin and levofloxacin/ciprofloxacin, respectively.

# Figure S6. Annual susceptibility of nonfermenting Gram-negative bacilli to piperacillin/tazobactam (A), ceftazidime (B), imipenem/cilastatin (C), amikacin (D) and ciprofloxacin (E).

Cochran-Armitage test p=0.23, p=0.781, p=0.141, p=0.696, p=0.289 for piperacillin/tazobactam, ceftazidime, imipenem/cilastatin, amikacin and ciprofloxacin, respectively.

# Figure S7. Annual susceptibility of enterococci to amoxicillin (A), gentamicin (B), and vancomycin (C).

Cochran-Armitage test p=0.955, p=0.054, p=0.083 for amoxicillin, gentamicin and vancomycin, respectively.

# Figure S8. Annual susceptibility of *Staphylococcus aureus* to oxacillin (A) gentamicin (B) ofloxacin/levofloxacin (C) and vancomycin (D).

Cochran-Armitage test p=0.229, p=0.790, p=0.060 and p=0.999 for oxacillin, gentamicin, ofloxacin/levofloxacin and vancomycin, respectively.

# Figure S9. Annual adequacy of EAT in the study population (A), in the group of patients with MDRO (B), and in the group of patients without MDRO (C).

Cochran-Armitage test, p=0.460, p=0.145 and p=0.0042, in the study population, patients with and without MDRO, respectively.

# Figure S10. Annual theoretical rate of adequacy of piperacillin/tazobactam (A), piperacillin/tazobactam + vancomycin (B), and imipenem/cilastatin + vancomycin (C) in patients with (red boxes) and without MDROs (open boxes).
